# Effect of Carbon Chain Length, Ionic Strength, and pH on the In Vitro Release Kinetics of Cationic Drugs from Fatty-Acid-Loaded Contact Lenses

**DOI:** 10.3390/pharmaceutics13071060

**Published:** 2021-07-10

**Authors:** Cesar Torres-Luna, Naiping Hu, Roman Domszy, Xin Fan, Jeff Yang, Robert M. Briber, Nam Sun Wang, Arthur Yang

**Affiliations:** 1 Department of Chemical & Biomolecular Engineering, University of Maryland, College Park, MD 20740, USA; cesartorres093@gmail.com; 2 Lynthera Corporation, 1200 Corporate Blvd., STE 10C, Lancaster, PA 17601, USA; naiping.hu@gmail.com (N.H.); rcdom@lynthera.com (R.D.); jeff@lynthera.com (J.Y.); 3 Department of Chemical Engineering, Auburn University, Auburn, AL 36849, USA; xzf0004@auburn.edu; 4 Department of Materials Science & Engineering, University of Maryland, College Park, MD 20740, USA; rbriber@umd.edu

**Keywords:** drug delivery, soft contact lenses, unsaturated fatty acid, cationic drug, oleic acid, sustained release, biomaterials

## Abstract

This paper explores the use of fatty acids in silicone hydrogel contact lenses for extending the release duration of cationic drugs. Drug release kinetics was dependent on the carbon chain length of the fatty acid loaded in the lens, with 12-, 14- and 18-carbon chain length fatty acids increasing the uptake and the release duration of ketotifen fumarate (KTF) and tetracaine hydrochloride (THCL). Drug release kinetics from oleic acid-loaded lenses was evaluated in phosphate buffer saline (PBS) at different ionic strengths (*I* = 167, 500, 1665 mM); the release duration of KTF and THCL was decreased with increasing ionic strength of the release medium. Furthermore, the release of KTF and THCL in deionized water did not show a burst and was significantly slower compared to that in PBS. The release kinetics of KTF and THCL was significantly faster when the pH of the release medium was decreased from 7.4 towards 5.5 because of the decrease in the relative amounts of oleate anions in the lens mostly populated at the polymer–pore interfaces. The use of boundary charges at the polymer–pore interfaces of a contact lens to enhance drug partition and extend its release is further confirmed by loading cationic phytosphingosine in contact lenses to attract an anionic drug.

## 1. Introduction

Ophthalmic diseases are most commonly treated with topical eye drops, and they comprise 90% of marketed ophthalmic formulations [1]. However, the active components from eye drops have less than 5% corneal bioavailability because of anatomical and physiological obstacles. Most of the applied drug components are drained from the eye via the lacrimal drainage system and ultimately absorbed systemically, where they carry the risk of inducing undesirable systemic allergic or pharmacological actions [2]. As a result, to attain the desired therapeutic drug concentrations, eye drops must be administered with frequent doses at drug concentrations significantly higher than the therapeutic dosage [3,4]. Moreover, patient compliance can be problematic with ophthalmic drops, especially among the elderly [5].

To overcome the drawbacks associated with conventional eye drop therapy, researchers have been trying to design more efficient delivery systems. An ideal drug delivery system is described as a system that delivers a required amount of drug to the ocular tissue with comfort, ease of administration, and no interference with vision or normal functioning of the eye [6]. Therapeutic contact lenses have been examined as drug delivery systems because of their unique properties like extended wear and over 50% drug bioavailability compared to eye drops [4,6,7]. The placement of a drug-eluting contact lens on the cornea separates the tear film into the pre-lens (exposed to the external environment) and the post-lens (between the lens and the cornea) compartments, reducing the drug load’s susceptibility to tear turnover as is common in eye drops [8]. The post-lens compartment’s relatively limited tear-mixing and exchange are favorable to prolonged contact time between the cornea and drug ingredient [9]. Consequently, a prolonged stay of the drug on the cornea leads to higher intraocular concentration, which results in improved pharmacological response compared with conventional topical administration [10]. Using contact lenses for drug delivery also may eliminate compliance issues related to a lack of consistency in the number and volume of drops and the precision of administration [11].

A simple method for delivering drugs by contact lenses is soaking the lenses in drug solution, followed by placement on the eye for release [12,13]. Unfortunately, release profiles are often characterized by an initial burst release followed by subtherapeutic dosing when delivery is more than one day [4,5,13]. The short duration of the release limits the potential benefits particularly for diseases that require consistently extended delivery over longer periods [13]. Recent methods to extend drug release from contact lenses have included the use of oil-in-water microemulsions [14,15,16], vitamin E diffusion barriers [17,18,19], micelles [20,21], liposomes [22,23], molecular imprinting [24], and ionic surfactants [25]. Such methods result in the release of drugs from contact lenses for extended periods of time, ranging from a few days to more than a month. Many of these technologies have addressed scientific and commercial challenges and are currently being tested in animal and clinical studies [13]. Therefore, it is likely that drug-eluting contact lenses will be commercialized in the near future [13,26].

The use of unsaturated fatty acids to extend the release duration of cationic drugs from commercial contact lenses has been recently reported by our group [27]. It was thought that electrostatic interactions at the interface between the hydrophobic domain and the aqueous pores of silicone hydrogel contact lenses were partially responsible for extending the release of cationic drugs. One aim of the current investigation is to further analyse these interfacial electrostatic interactions to study the effect of the carbon chain length of the fatty acid on the release kinetics of tetracaine hydrochloride (THCL) and ketotifen fumarate (KTF). These ophthalmic drugs are positively charged at physiological pH. The release studies were performed in daily disposable silicone hydrogel contact lenses and therefore, the release kinetic profiles were accommodated to the intended modality of these lenses. Because the interfacial properties of fatty acids and the lipids derived from them markedly depend on their carbon chain length [28], it was hypothesized that medium-chain and long-chain fatty acids would have a different impact on the drug release kinetics.

The second aim of this study is to investigate the influence of ionic strength of the release medium on the drug release kinetics from fatty acid-loaded lenses. For drugs that are charged at physiological pH, it is expected that the salt composition of the release medium will have a significant impact on their release from contact lenses [29]. It is predicted that the effect of electrostatic interactions between charged drugs and oppositely charged contact lenses is diminished with increasing ionic strength of the release media. To confirm these premises, the in vitro release kinetics of KTF and THCL in phosphate buffer saline (PBS) media was evaluated under three different ionic strength (*I* = 167, 500, 1665 mM). The drug release in PBS was also compared to the drug release in the absence of added ions (i.e., in deionized water). Finally, the impact of the pH of the release medium on the drug release kinetics was also assessed.

As the pH of the release medium is varied, the relative amounts of oleate anions in the lens and charge density of the drug are expected to change. To confirm this, the release kinetics at physiological pH was compared to that in the medium at pH values of 6.4 and 5.5. The results as described in this study help to explain the dominant mechanisms behind the release extension of cationic drugs from fatty acid-loaded contact lenses. Last, but not least, a cationic surface active agent, phytosphingosine, was incorporated in contact lenses and demonstrated the same electrostatic control of releasing the oppositely charged anionic drugs.

## 2. Materials and Methods

### 2.1. Materials

Two commercial silicone hydrogel contact lenses (diopter -3.50) (ACUVUE TruEye^®^ and Dailies Total1^®^) are used in this study. The detailed information of these commercial lenses is described in Table 1. Tetracaine hydrochloride (THCL) pharmaceutical secondary standard, ketotifen fumarate (KTF) salt, Dulbecco’s Phosphate Buffered Saline (PBS) 1×, Dulbecco’s Phosphate Buffered Saline 10×, octanoic acid (≥99%), capric acid (≥98%), lauric acid (≥99%), myristic acid (≥99%) were purchased from Millipore Sigma (Millipore Sigma Corp, St. Louis, MO, USA). Oleic acid (90%) was purchased from Alfa Aesar (Tewksbury, MA, USA). Ethanol (≥ 99.5%) was purchased from Pharmco (Brookfield, CT, USA). Phytosphingosine (≥95%) was purchased from Cayman Chemical Company (Ann Arbor, MI, USA).

### 2.2. Fatty-Acid-Loading into Pristine Silicone Hydrogel Contact Lenses

ACUVUE TruEye^®^ and Dailies Total1^®^ contact lenses were rinsed with deionized water and then air-dried overnight before use. Dried contact lenses were soaked in 4 mL of 25 mM of fatty acid in ethanol. The fatty acids tested were oleic acid, myristic acid, lauric acid, capric acid, and octanoic acid. Only for oleic acid, a fatty acid soaking concentration of 50 mM was also tested. The soaking duration was 24 h at room temperature. Following the loading step, the contact lenses were taken out and washed in deionized water for 1 h and subsequently air-dried overnight.

### 2.3. Phytosphingosine-Loading into Pristine Silicone Hydrogel Contact Lenses

ACUVUE TruEye^®^ contact lenses were rinsed with deionized water and then air-dried overnight before use. Dried contact lenses were soaked in 4 mL of 10 mM or 20 mM of phytosphingosine in ethanol. The soaking duration was 24 h at room temperature. Following the loading step, the contact lenses were taken out and washed in deionized water for 1 h and subsequently air-dried overnight.

### 2.4. Drug-Loading into Fatty-Acid-Loaded Lenses or Phytosphingosine-Loaded Lenses

Drugs were loaded into the control and the fatty-acid-loaded contact lenses by soaking in 5 mL of drug-loading solutions. THCL was loaded at a concentration of 0.2 mg/mL of drug in phosphate buffer saline (PBS), KTF was loaded at 0.3 mg/mL, and diclofenac sodium (DFNa) was loaded at 0.2 mg/mL. The duration of the soaking was 1 day at room temperature. Following the loading period, the lenses were taken out and excess drug solution on the surface was removed by blotting with Kimwipes. To determine the amount of drug loaded in each lens, the drug concentration of each soaking solution was measured before and after the soaking period using a UV-visible spectrophotometer (Varian Cary 50 Bio, Walnut Creek, CA, USA). After the drug-loading step, lenses were tested using in vitro release experiments.

### 2.5. Drug Release Experiments

The drug release experiments were carried out by soaking the drug loaded lenses in 3 mL PBS (pH 7.4) at room temperature. During the release experiments, 1 mL of the release sample was removed at predetermined time intervals, and 1 mL of fresh PBS was refilled into the release medium. The drug concentration at each time interval was measured using a UV-Spectrophotometer (Varian Cary 50 Bio) at wavelengths of 315 nm for THCl, 300 nm for KTF, and 276 nm for DFNa. The drug release experiments were performed in triplicate for each different case. Control contact lenses having fatty acid, but no drug were also tested to account for absorbance without the presence of drug.

#### 2.5.1. Effect of Ionic Strength in Release Medium on Drug Release Kinetics

To investigate the effect of electrostatic interactions on drug release kinetics, the release experiments were conducted at three different ionic strengths. In these experiments, two different PBS from Millipore Sigma with ionic strengths of 167 mM (1× PBS) and 1665 mM (10× PBS) were used. PBS having an ionic strength of 500 mM was prepared by diluting 10× PBS with deionized water. For all experiments, drug-loading was performed using the same PBS (i.e., 1× PBS).

#### 2.5.2. Effect of pH of Release Medium on Drug Release Kinetics

The effect of pH of the release medium on the drug release kinetics was studied at 3 different pH values. In these experiments, the pH of PBS (1×, pH = 7.4) was lowered by adding HCl (1.0 M). The release studies were conducted at three different pH values: 7.4, 6.4 and 5.5.

## 3. Results and Discussion

### 3.1. Effect of Fatty Acid Carbon Chain Length on Release Kinetics of Cationic Drugs

**Figure 1:**
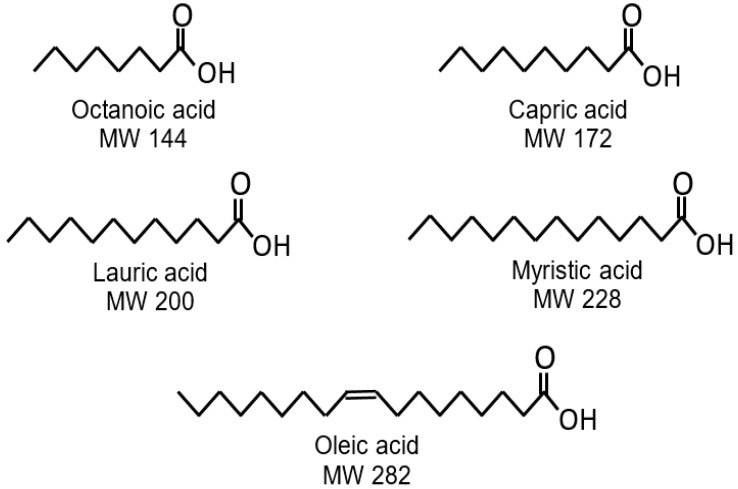
Molecular structure of the fatty acids tested in the current study.

The properties of fatty acids and their lipid derivatives, particularly at a hydrophobic–hydrophilic interface, markedly depend on hydrocarbon chain length [28]. Thus, the length of the hydrophobic chain may have a direct impact on the anionic dissociation of fatty acids. In the present study, we tested five fatty acids with a different number of carbon atoms to evaluate the impact of hydrocarbon chain length on the uptake and release kinetics of tetracaine hydrochloride (THCL) and ketotifen fumarate (KTF). Figure 1 shows the molecular structure, and Table 2 summarizes the properties of the tested fatty acids. Oleic acid, myristic acid, lauric acid, capric acid, and octanoic acid have an 18, 14, 12, 10, and 8-carbon chain length, respectively. Octanoic acid, capric acid, and lauric acid are considered medium-chain fatty acids, while myristic acid and oleic acid are considered long-chain fatty acids [30].

As shown in Table 2, the aqueous solubility of a fatty acid exponentially decreases with an increase in the carbon chain length. Because of hydrocarbon chains being nonpolar, fatty acids having long hydrocarbon chains are mainly hydrophobic despite having one polar functional group. For instance, the water solubility of octanoic acid (C8) is over three orders of magnitude greater than that of myristic acid (C14) because of the increase in the aliphatic carbon chain. However, for fatty acids dissolved in the hydrophobic silicone domain of contact lenses or adsorbed at the silicone–water interfaces, we expect that the enhanced hydrophobic bonding from longer hydrocarbon chains to increase their solubility in the silicone domains and increase packing densities at the interface. The length of the tail section of the fatty acid (or another surface active agent) needs to be sufficiently long to create a high loading density at the polymer–aqueous interfaces through stronger adsorption and hydrophobic bonding to the gel matrix [25]. The high packing density and hydrophobic affinity will also minimize the potential release of the fatty acid into the tears along with the intended drug delivery release [25]. If a long-chain fatty acid like oleic acid packs at a high ligand density at the interface (attributed to a higher loading % in the bulk polymer), the charge density can be appreciable for increasing uptake and release duration of oppositely charged drugs.

**Figure 2:**
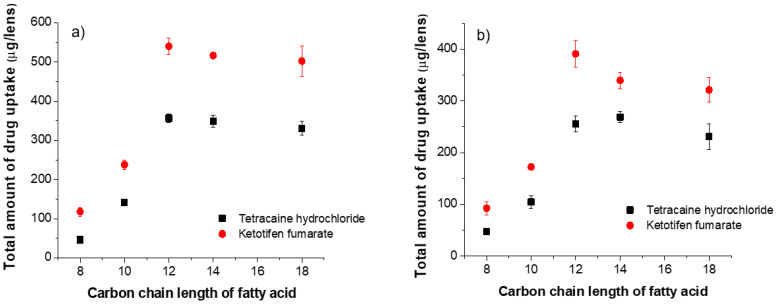
The total amount of drug uptake as a function of carbon chain length of fatty acid. Fatty acids are loaded in silicone hydrogel commercial lenses at a fixed concentration of 25 mM. (**a**) ACUVUE TruEye^®^. The total amount of drug uptake for control lenses (i.e., without fatty acid) is: 120.3 ± 4.0 µg for THCL and 211.1 ± 13.0 µg for KTF. (**b**) Dailies Total1^®^. The total amount of drug uptake for control lenses (i.e., without fatty acid) is: 99.4 ± 1.7 µg for THCL and 152.8 ± 7.1 µg for KTF.

**Figure 3:**
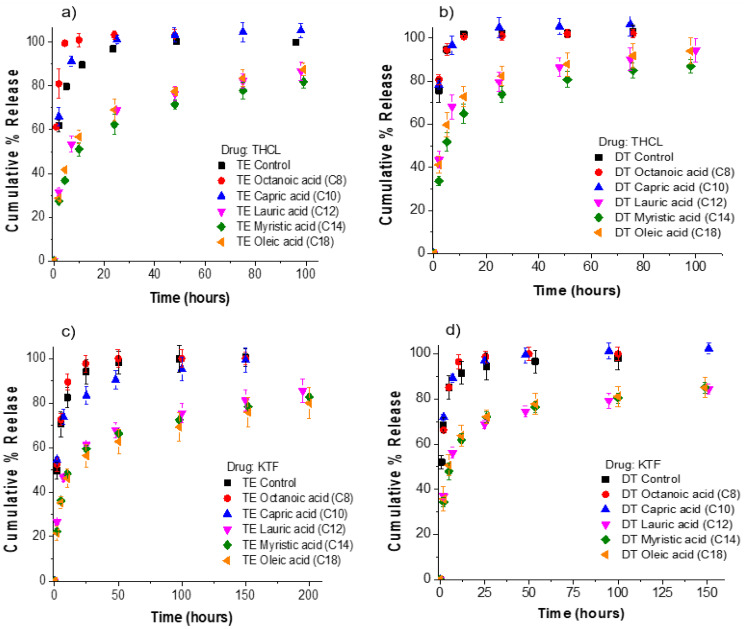
Cumulative % release of KTF and THCL from silicone hydrogel commercial lenses. Fatty acids in contact lenses were loaded at a fixed soaking concentration of 25 mM. The total amount of drug uptake for each fatty-acid-loaded lens case is displayed in Figure 2. (**a**) THCL cumulative % release from ACUVUE TruEye^®^. The total amount of drug uptake: 120.3 ± 4.0 µg for control lenses. (**b**) THCL cumulative % release from Dailies Total1^®^. The total amount of drug uptake: 99.4 ± 1.7 µg for control lenses. (**c**) KTF cumulative % release from ACUVUE TruEye^®^. The total amount of drug uptake: 211.1 ± 13.0 µg for control lenses. (**d**) KTF cumulative % release from Dailies Total1^®^. The total amount of drug uptake: 152.8 ± 7.1 µg for control lenses Data are presented as mean ± standard deviation with *n* = 3.

Figure 2 shows the total amount of drug uptake in ACUVUE TruEye^®^ and Dailies Total1^®^ lenses as a function of the carbon chain length of the fatty acid loaded in the lens. The drug uptake was substantially increased when the chain length of the fatty acid was longer than 10. For fatty acids with shorter chain length (C8 and C10) their high aqueous solubilities led to a much lower population density at the pore surface and thus, limited their abilities in generating interfacial anionic boundary charges. The substantial increase in cationic drug uptake with fatty acids having a chain length longer than 12 shows enhanced drug affinity at pore interfaces because of more anionic boundary charges. As expected, it significantly extended the cationic drug release kinetics as shown in Figure 3.

The release kinetics of THCL and KTF from ACUVUE TruEye^®^ and Dailies Total1^®^ contact lenses is shown in Figure 3. We define the release duration as the time for 70% cumulative drug release. For THCL in TruEye^®^, control lenses and lenses loaded with either octanoic acid or capric acid have release durations of less than 4 h. Moreover, TruEye^®^ lenses loaded with oleic acid, myristic acid, or lauric acid have release durations of greater than 24 h. For the case of THCL in Dailies Total1^®^, control lenses and lenses loaded with either octanoic acid or capric acid have release durations of less than 2 h.

Dailies Total1^®^ lenses loaded with oleic acid, myristic acid, or lauric acid have release durations of greater than 8 h. For KTF in TruEye^®^, control lenses and lenses loaded with octanoic acid or capric acid have release durations of less than 5 h. TruEye^®^ lenses loaded with oleic acid, myristic acid, or lauric acid have release durations of greater than 48 h. For Dailies Total1^®^, control lenses and lenses loaded with octanoic acid or capric acid have release durations of less than 3 h. Dailies Total1^®^ lenses with oleic acid, myristic acid, or lauric acid have release durations of at least 20 h. For both contact lenses and both cationic drugs, the release kinetic studies showed that oleic acid, myristic acid, and lauric acid could significantly increase the release duration of KTF and THCL, while octanoic acid and capric acid did not impact either drug. Oleic acid, with 18 carbon and the lowest aqueous solubility among all, is the most ideal for controlling cationic drug release from fatty-acid-loaded contact lenses because of generating a high boundary charge density and potentially not leaching out with the drug during the lens shelf storage or in use.

The goal of this study is to use a fatty acid soaking concentration to achieve drug release duration within the intended use of the contact lenses. ACUVUE TruEye^®^ and Dailies Total1^®^ are daily disposable silicone hydrogel contact lenses. Therefore, we targeted release durations between 8 and 24 h that are consistent with the daily wear schedule of the lenses. Table 3 summarizes the release durations that were obtained for control lenses and lenses loaded with fatty acids. Dailies Total1^®^ lenses loaded with oleic acid, lauric acid, or myristic acid have release durations between 8–24 h for both cationic drugs. For ACUVUE TruEye^®^, lenses loaded with oleic acid, lauric acid, or myristic acid achieve release durations between 8–24 h for THCL. 

However, in the case of KTF, release durations between 60–100 h are achieved. Therefore, only for the case of KTF release from TruEye^®^ the fatty acid soaking concentration would need to be slightly decreased to achieve release durations within the daily wear schedule of the lens. Ketotifen fumarate is an antihistamine drug for the treatment of allergic conjunctivitis; however, it has been reported that it is essential to maintain a stable ketotifen concentration in the tear fluid for a long time for an effective treatment against allergic conjunctivitis [34]. Because of the higher drug bioavailability that is achieved from a drug-eluting contact lens, this could provide an effective therapy by delivering sufficient concentrations of ketotifen to the tear fluid.

For instance, a contact lens-based delivery system for ketotifen was tested in two parallel, conjunctival allergen challenge-based trials, and the system was reported to be the first demonstration of efficacy for contact lens delivery of a therapeutic for ocular allergy and further suggested that it can provide a means of simultaneous vision correction and treatment for contact lens wearers with ocular allergies [35]. In the case of tetracaine hydrochloride, it is a topical anesthetic drug used after ocular surgical procedures such as photorefractive keratectomy to control pain after the surgery [36]. It has been reported that at high dosage concentrations (i.e., above 0.3125 g/L), THCL has a dose and time-dependent cytotoxicity to human corneal epithelial cells in vitro [37]. Therefore, achieving controlled release of THCL by using contact lenses loaded with fatty acids can be considered as a potential alternative to deliver THCL with precise dosages, minimized systemic effects, and with no preservatives to the ocular tissues to avoid detrimental side effects.

### 3.2. Effect of Ionic Strength of Release Medium on Release Kinetics

**Figure 4:**
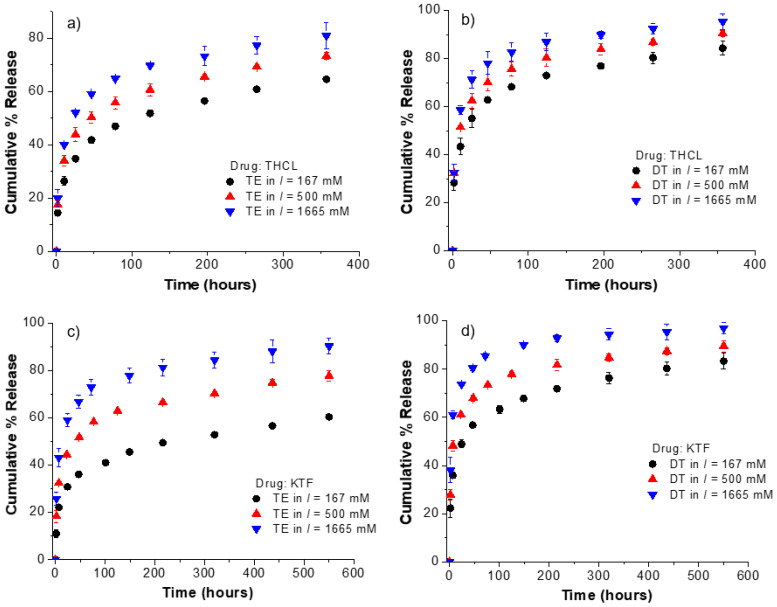
Effect of the ionic strength of the release medium on the cumulative % release kinetics from ACUVUE TruEye^®^ and Dailies Total1^®^ loaded with oleic acid. Oleic acid was loaded at a fixed soaking concentration of 50 mM for all cases. (**a**) THCL cumulative % release from ACUVUE TruEye^®^. The total amount of drug uptake: 539.4 ± 11.8 µg. (**b**) THCL cumulative % release from Dailies Total1^®^. The total amount of drug uptake: 362.4 ± 30.3 µg. (**c**) KTF cumulative % release from ACUVUE TruEye^®^. The total amount of drug uptake: 781.0 ± 33.6 µg. (**d**) KTF cumulative % release from Dailies Total1^®^. The total amount of drug uptake: 528.9 ± 31.0 µg. Data are presented as mean ± standard deviation with *n* = 3.

Because THCL and KTF are charged molecules at physiological pH, their interfacial partitioning and diffusive transport are expected to be affected by the ionic strength of the release medium. To confirm whether electrostatic interactions play a significant role in the drug adsorption and transport from fatty-acid-loaded lenses, the release kinetics of KTF and THCL was investigated in PBS solutions with different ionic strength (*I* = 167, 500, 1665 mM). For both ACUVUE TruEye^®^ and Dailies Total1^®^, an oleic acid soaking concentration of 50 mM was used. At this concentration, the oleic acid weight % was 2.5 and 5.2% (weight of oleic acid/weight of dry unmodified lens) for ACUVUE TruEye^®^ and Dailies Total1^®^, respectively. Drug-loading was done at a fixed concentration, time, and volume media using the conventional PBS (i.e., *I* = 167 mM) for all cases. Under these conditions, ACUVUE TruEye^®^ lenses with 2.5% OA uptake approximately 540 µg and 780 µg of THCL and KTF, respectively, while the Dailies Total1^®^ lenses with 5.2% OA uptake 360 µg and 530 µg of THCL and KTF, respectively. The release kinetics of KTF and THCL is shown in Figure 4. An increase in the ionic strength of the release medium leads to a more accelerated release for both cationic drugs. For instance, the release duration of KTF from Dailies Total1^®^ lenses loaded with oleic acid is 200 h, 75 h, and 15 h for *I* = 167, 500, and 1665 mM, respectively. A similar trend is observed for ACUVUE TruEye^®^ lenses and for THCL.

The results show that the effect of electrostatic interactions is significantly reduced with increasing ionic strength, therefore leading to faster release kinetics. In another study, Kim et al. evaluated the transport of three derivatives of dexamethasone from poly (hydroxyethyl methacrylate) (pHEMA) contact lenses [29]. The ionic strength in the release solution had a negligible effect on the transport of the two non-ionic derivatives of dexamethasone; however, the drug transport of the ionic derivative of dexamethasone (i.e., dexamethasone 21-disodium phosphate) depended on the ionic strength of the release medium [29]. A different study reported that the ionic strength of the drug-loading solution significantly affects the equilibrium partition coefficient of anionic diclofenac sodium in both pHEMA and silicone hydrogel contact lenses [38]. Zhu et al. [39] also reported the increase of diclofenac sodium uptake in pHEMA contact lenses when using low ionic strength solutions for drug-loading. However, it should be noted that the mentioned studies used unmodified contact lenses (i.e., without ionic co-monomers or ionic surface-active agents).

In the present study, the impact of ionic strength becomes more significant because of the addition of the fatty acid anionic charges in the contact lenses, which create strong electrostatic interactions with the cationic drugs that can be remarkably manipulated when adjusting the ionic strength of either drug-loading or releasing medium.

From Figure 4, it was observed that drug release duration is more extended in ACUVUE TruEye^®^ compared to Dailies Total1^®^ even though the latter absorbs twice as much oleic acid as the former, as mentioned earlier. These results agree with the higher bulk water content of ACUVUE TruEye^®^ compared to Dailies Total1^®^, as shown in Table 1. In our previous study, ACUVUE TruEye^®^ and ACUVUE Oasys^®^ loaded with oleic acid showed a similar trend [27]: TruEye^®^ lenses with oleic acid extended the release of cationic drugs longer than ACUVUE Oasys^®^ lenses because of the higher water content of the former (i.e., 46% for TruEye^®^ and 38% for Oasys^®^).

For silicone hydrogel contact lenses, smaller pores are associated with low water content in the silicone phase [40]. Since electrostatic interactions take place at the interfaces of the hydrophobic silicone domain and the aqueous pores of the contact lenses, higher water content is associated with a higher number of oleate anions at the interfaces and hence, a more extended drug release. Dailies Total1^®^ is a “water-gradient” contact lens that comprises an 80-µm silicon hydrogel core with approximately 33% water content surrounded by a 10-µm-high water content layer of >80% [41,42]. As a result, due to its lower water content, the burst release of THCL and KTF is higher in Dailies Total1^®^ than in ACUVUE TruEye^®^.

In addition, the thin high water content surface layer of Dailies Total1^®^ has been demonstrated to increase the burst release for hydrophilic drugs [41]. Ophthalmic drugs such as THCL and KTF are hydrophilic at physiological pH because of the charge [36], which also explains the significant burst release observed for Dailies Total1^®^ compared to ACUVUE TruEye^®^. However, even though ACUVUE TruEye^®^ has a higher bulk water content of 46% that helps to prevent a high burst release, Dailies Total1^®^ has a much higher oxygen permeability and enhanced surface wettability because of the highly lubricious surface layer provided by its water-gradient feature [43].

**Figure 5:**
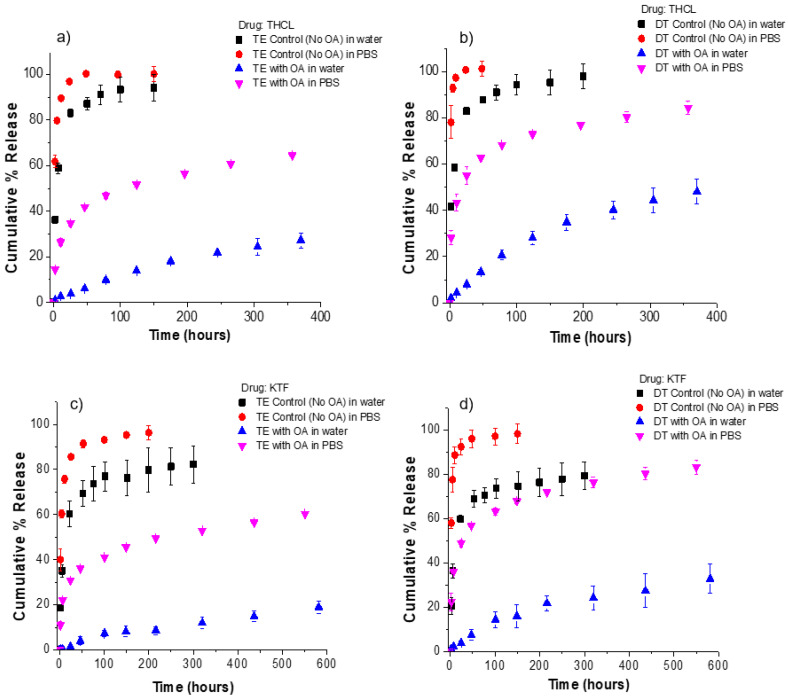
Release kinetics of KTF and THCL from unmodified and oleic acid loaded ACUVUE TruEye^®^ and Dailies Total1^®^ in deionized water or 1× PBS. Oleic acid was loaded at a fixed soaking concentration of 50 mM for all cases. (**a**) THCL cumulative % release from ACUVUE TruEye^®^. The total amount of drug uptake: 120.3 ± 4.0 µg for control lenses and 539.6 ± 3.0 µg for OA lenses. (**b**) THCL cumulative % release from Dailies Total1^®^. The total amount of drug uptake: 99.4 ± 1.7 µg for control lenses and 360.8 ± 13.4 µg for OA lenses. (**c**) KTF cumulative % release from ACUVUE TruEye^®^. The total amount of drug uptake: 211.1 ± 13.0 µg for control lenses and 802.3 ± 7.0 µg for OA lenses. (**d**) KTF cumulative % release from Dailies Total1^®^. The total amount of drug uptake: 152.8 ± 7.1 µg for control lenses and 516.8 ± 70.6 µg for OA lenses. Data are presented as mean ± standard deviation with *n* = 3.

For the case of drugs that are expected to be charged at pH values between 7.0 and 7.5 (i.e., physiological pH), the release kinetics behavior is expected to be significantly different in deionized water from that in PBS [29]. Because of this, the release kinetics of THCL and KTF from unmodified and oleic-acid-loaded contact lenses was studied in deionized water and compared to that in conventional PBS. The oleic acid soaking concentration and drug-loading conditions were kept the same as with the ionic strength study. For unmodified lenses (i.e., without oleic acid), the release durations of THCL in either PBS or deionized water are less than 24 h for both ACUVUE TruEye^®^ and Dailies Total1^®^ as shown in Figure 5.

In the case of KTF, unmodified lenses have release durations of less than 48 h (in water or PBS). The release kinetics of THCL and KTF from unmodified contact lenses demonstrated the typical nonlinear kinetics: a burst of drug released during the first few hours, followed by declining, subtherapeutic levels of drug release in the later stages [5]. However, for oleic-acid-loaded lenses in water, there is an absence of the typical burst release stage in the first 24 h. For both THCL and KTF, less than 10% of loaded drug is released after 24 h from TruEye^®^ and Dailies Total1^®^. In fact, TruEye^®^ lenses loaded with oleic acid release THCL and KTF at a nearly constant rate for 400 and 600 h, respectively. It should be noted that the extended release was obtained for lenses loaded with ~5% weight of oleic acid. In our prior studies, oleic acid weight percentages as high as 28.6% were used, and the release kinetics strongly depended on the weight % of oleic acid in the contact lenses [27]. Therefore, by increasing the oleic acid weight % in the lenses, the release of KTF and THCL in water could be further prolonged if desired.

One of the main commercialization challenges for drug-eluting contact lenses is the drug’s premature release during storage in a packaging solution [6,13]. All commercial contact lenses must be shipped and stored in a wet medium to avoid becoming brittle [44]. Stimulus-triggered drug release from contact lenses is an emerging technique to deliver therapeutic payloads on demand while preventing drug loss because of premature elution from the lenses during shipping and storage [45]. Researchers have explored temperature [45], pH [46], and biological [47] stimuli-triggered delivery systems in contact lenses to avoid drugs to elute prematurely. In the present study, the strong dependence of charged drug release on ionic strength offers a solution to avoid the drug’s premature discharge during storage of fatty-acid-loaded contact lenses in a packaging solution. For instance, oleic-acid-loaded lenses containing KTF or THCL could be stored in low ionic strength solution media (i.e., 0–100 mM). When such drug-eluting contact lenses are placed on the eye, the tear fluid (~150–200 mM) will slowly diffuse inside the lens matrix and make the drug molecules to be released from the lens due to equalizing ion-exchange process.

Drug transport in PBS, which is a reasonable mimic of the tear fluid, may differ from that in deionized water because of differences in drug binding to the lens in the two mediums or different degrees of swelling of the gels [29]. Even though the different degree of swelling of the lenses in PBS and water could have also influenced the release kinetics of KTF or THCL, researchers have reported that changing the medium from water to PBS has led to an increase in the equilibrium water content of pHEMA and silicone hydrogel lenses smaller than 3% [38]. Therefore, the different degrees of swelling of TruEye^®^ or Dailies Total1^®^ in water and PBS could not account for the significant difference in the release kinetics from the oleic acid-loaded lenses in water and PBS. This is further confirmed by the release kinetic profiles from the unmodified control lenses: as shown in Figure 5, when using either PBS or deionized water as the release medium, the release durations of THCL and KTF from the unmodified lenses are less than 24 h and 48 h, respectively. Therefore, these results confirm the significant role of electrostatic interactions between the oleate anions in the lens and the loaded cationic drugs on the release extension. In one study, Lee et al. evaluated the release of the cationic drug ofloxacin from negatively charged co-monomers in pHEMA contact lenses [48]. They concluded that the perturbation of electrostatic interactions because of using a salt-dissolved solution for drug-loading caused a significant decrease in the amount of ofloxacin released from the lenses. Furthermore, the authors reported that loading in PBS buffer containing ofloxacin led to the release of 35 µg from the negatively-charged lenses, whereas loading in ofloxacin aqueous solution resulted in the release of 341 µg from the same lens [48]. Similarly, higher amounts of THCL or KTF can be loaded in oleic-acid-loaded lenses using a low ionic strength solvent.

With the purpose of demonstrating the application of boundary charge modifiers to increase the release duration of anionic drugs as well, we loaded phytosphingosine in ACUVUE TruEye^®^ contact lenses. Phytosphingosine is a naturally occurring lipid having an 18-carbon chain length. It is cationic at neutral pH [49] and, therefore, can extend the release of anionic drugs when loaded in contact lenses. Because it is practically insoluble in water, it must be loaded in contact lenses by ethanol solvent soaking as in the case of oleic acid.

**Figure 6:**
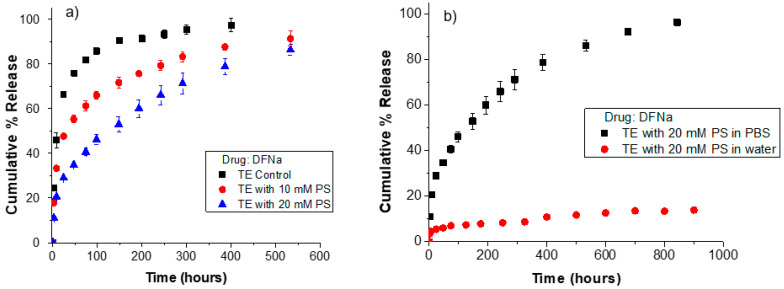
Release kinetics of DFNa from unmodified and phytosphingosine (PS) loaded ACUVUE TruEye^®^. (**a**) DFNa cumulative % release in PBS from ACUVUE TruEye^®^. The total amount of drug uptake: 219.8 ± 3.2 µg for control lenses, 381.5 ± 7.4 µg for TruEye^®^ with 10 mM PS, and 567.8 ± 9.5 µg for TruEye^®^ with 20 mM PS. (**b**) DFNa cumulative % release in PBS or deionized water from ACUVUE TruEye^®^ lenses loaded with 20 mM PS. The total amount of drug uptake: 567.8 ± 9.5 µg for TruEye^®^ lenses loaded with 20 mM PS.

Diclofenac sodium (DFNa), an anionic non-steroidal anti-inflammatory drug, was used as the model drug. DFNa has a pKa value of ~4, and it is dissociated to more than 99% and exhibits a negative charge at physiological pH [50]. Figure 6 displays the release kinetics of DFNa from TruEye^®^ control lenses and TruEye^®^ lenses loaded with phytosphingosine (PS). As shown, control lenses have a release duration of 40 h while lenses loaded with 10 mM and 20 mM PS have release durations of 130 h and 275 h, respectively. These results show that PS in TruEye^®^ lenses substantially extends the release kinetics of anionic DFNa because of electrostatic interactions.

The release in deionized water of lenses loaded with PS is significantly slower than the release in PBS and lacks an initial burst stage as in the release in water from oleic-acid-loaded lenses. For instance, lenses loaded with 20 mM PS release in water less than 10% of DFNa after 200 h, while lenses in PBS release 60% in this period. After 900 h, lenses with 20 mM PS release less than 15% of the drug in deionized water, while the release in PBS reaches over 90%. These results show both the high surface charge density of PS at the pore surface of the TruEye^®^ contact lenses and the dependence of DFNa release kinetics from PS loaded lenses on the ionic strength of the release medium. Unlike oleic acid, PS is commercially available as a crystalline solid and therefore, its loading in contact lenses may be limited to lower amounts to prevent aggregation. Our experiments showed that loadings above 20 mM PS compromised the optical transparency of the contact lenses. Nevertheless, the results showed that even loading at a dosage lower than 20 mM, PS can still significantly extend the release of the anionic drug compared to pristine lenses.

### 3.3. Effect of pH of Release Medium on Release Kinetics

**Figure 7:**
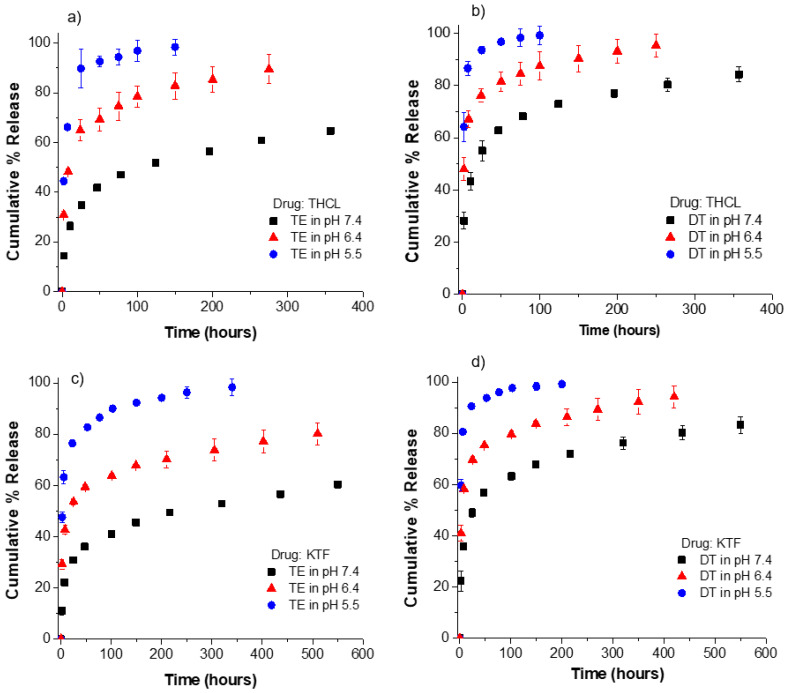
Release kinetics of KTF and THCL from oleic acid-loaded ACUVUE TruEye^®^ and Dailies Total1^®^ in PBS at different pH values (5.5, 6.4, and 7.4). Oleic acid was loaded at a fixed soaking concentration of 50 mM for all cases. (**a**) THCL cumulative % release from ACUVUE TruEye^®^. The total amount of drug uptake: 576.3 ± 9.6 µg for OA lenses. (**b**) THCL cumulative % release from Dailies Total1^®^. The total amount of drug uptake: 389.6 ± 20.0 µg for OA lenses. (**c**) KTF cumulative % release from ACUVUE TruEye^®^. The total amount of drug uptake: 813.5 ± 5.0 µg for OA lenses. (**d**) KTF cumulative % release from Dailies Total1^®^. The total amount of drug uptake: 531.6 ± 4.0 µg for OA lenses. Data are presented as mean ± standard deviation with *n* = 3.

Because ionic strength had a significant impact on the drug release kinetic profiles from oleic acid-loaded contact lenses, it was expected that the pH of the release medium would also strongly affect the release kinetics of KTF or THCL. To evaluate the effect of pH, we examined the drug release kinetics in PBS under three different pH values (5.5, 6.4, and 7.4). The pH of the release medium was varied between 5.5 and 7.4 to simulate the pH range of commercial saline solution [51] and lens packaging solution [46]. ACUVUE TruEye^®^ and Dailies Total1^®^ lenses were soaked in an oleic acid soaking concentration of 50 mM; at this concentration, the oleic acid weight% was 2.5% and 5.2% for ACUVUE TruEye^®^ and Dailies Total1^®^, respectively. The drug-loading conditions were kept the same for all oleic acid-loaded contact lenses. As shown in Figure 7, the pH of the release medium significantly affects KTF and THCL release kinetics from the oleic acid-loaded lenses. For example, the release durations of THCL from TruEye^®^ with oleic acid in PBS at a pH of 5.5 and 6.4 are 10 h and 50 h, respectively. On the other hand, the release durations in PBS at the physiological pH are greater than 300 h. For the case of Dailies Total1^®^ with oleic acid, THCL release durations are 3 h, 10 h, and 100 h in PBS at a pH of 5.5, 6.4, and 7.4, respectively. The release kinetics of KTF is also accelerated as the pH of the release medium decreases from 7.4 to 5.5.

The obtained results demonstrate the pH-responsiveness feature of oleic acid-loaded contact lenses. Prior studies have reported the use of fatty acids for the synthesis of pH-responsive nanostructured lipid carriers [52,53,54]. Chen et al. [54] reported using pH-responsive solid lipid nanoparticles based on sodium laurate and doxorubicin to improve the efficacy of multidrug resistance cancer chemotherapy. The study showed that the solid lipid nanoparticles exhibited pH-dependent drug release behaviors, with the highest drug release at pH 4.7 and the lowest drug release at pH 7.4 [54]. The authors postulated that the release of cationic doxorubicin at lower pH was facilitated by the protonation of the carboxyl group of laurate, leading to the reduction of the electrostatic attractions between the negatively charged laurate and the positively charged doxorubicin. Similarly, in the present study, when the pH of the release medium decreases from 7.4 to 5.5, this leads to the protonation of the oleic acid carboxylates and subsequent rapid release of the cationic drug molecules from the lenses.

Moreover, the faster release at lower pH could be also attributed to the increase in the solubility of the drugs. For example, the solubility in PBS of ketotifen fumarate at pH 7 and 10 was found to be 10.5 and 0.02 mg/mL, respectively [55]. The degree of ionization of KTF and THCL increases at lower pH values, which leads to higher aqueous solubilities in more acidic pH environments.

Similar to the previously observed ionic strength effects on kinetics, the dependence of cationic THCL and KTF release kinetics on the pH of the release medium can also be used for avoiding significant amounts of drug loss during lens packaging and storage. There have been studies that reported the application of pH-triggered drug release from contact lenses [44,46]. For example, Maulvi et al. [46] reported pH-sensitive nanoparticles in contact lenses to achieve sustained release of cyclosporine at therapeutic rates without leaching of drug during sterilization and storage period. It was reported that the nanoparticles did not allow the entrapped drug to release in packaging solution (pH 6.5) for 3 months, and therefore patients can use the developed contact lenses after removing from the packaging solution (pH < 7) and washing it with normal saline (pH = 7.4) prior to use. In our studies, we can also manipulate drug leaching by controlling the pH of the storage and release media.

However, based on the results presented in Figure 5 and Figure 7, the most significant effect to reduce drug leaching would come from using a packaging solution medium having a low ionic strength. The use of a simple solution loading of fatty acids, or other charged surface-active modifiers, to control the release of oppositely charged cargo molecules represents one of the easiest and most versatile tools to modify contact lenses for drug delivery applications. This approach is straightforward and can be incorporated into current contact lens’ manufacturing processes with minimum concerns in fabrication and scale-up costs. Furthermore, the use of variation in ionic strength to control the triggering or fine-tuning drug release kinetics is user-friendly and safe as long as the storage medium is low in ionic strength but similar in osmolarity (isotonic) to tears.

## 4. Conclusions

This study showed that fatty acids in silicone hydrogel commercial contact lenses can increase the drug release duration of cationic drugs such as tetracaine hydrochloride and ketotifen fumarate. Drug uptake and release depended on the carbon chain length of the fatty acid loaded in the contact lens. Lauric acid, myristic acid, and oleic acid having a 12, 14, and 18 carbon chain length, respectively, significantly increased drug release duration of KTF and THCL. The drug release durations (70% drug released) reached with the fatty-acid-loaded lenses were between 8 h and 24 h, which are consistent with the daily wear schedule of the commercial lenses used in the study. The release duration can be further extended by adjusting the fatty acid weight % in the lens. The release kinetics of KTF and THCL from oleic acid-loaded lenses was influenced by the ionic strength of the release medium; drug release was accelerated with increasing ionic strength because of the increased shielding of electrostatic interactions between the drugs and the oleic-acid-loaded interfaces. The release of KTF and THCL from oleic-acid-loaded lenses in deionized water did not show a burst and followed a nearly constant release rate, particularly for ACUVUE TruEye. The pH of the release medium also affected drug release; decreasing the pH accelerated release kinetics. The biocompatible nature of fatty acids with the ocular surface is an advantage for ocular drug delivery devices, including extended cationic drug release kinetics and reduction of premature discharge from a CL device as reported in this work. Finally, a similar effect was verified by using phytosphingosine as a cationic boundary charge modifier to control the release of anionic drug DFNa. The boundary charge modified CLs can become a platform delivery vehicle for controlling release of various charged cargo molecules of proteins, peptides, or nanocarriers. Prior to cytotoxicity evaluation and animal studies of this drug delivery platform, several key material properties of the lenses, such as their oxygen permeability, wettability, and modulus, will be evaluated.

## Figures and Tables

**Table 1:** List of silicone hydrogel commercial contact lenses that are used in the present study.

Commercial Name	Material	Manufacturer	Water Content (%)	Modulus (MPa)	Center Thickness (mm)
ACUVUE TruEye^®^	Narafilcon A	Johnson & Johnson Vision Care	46%	0.66	0.09
Dailies Total1^®^	Delefilcon A	Alcon Inc.	33% (bulk) ≥80% (surface)	0.7	0.09

**Table 2:** Physical and solution properties of the studied fatty acids.

Fatty Acid	# of Carbons and Degree of Saturation	Melting Point ^a^ (C)	Solubility in Water ^b^ (M)
Octanoic acid	8:0	16–17	4.7 × 10^−3^
Capric acid	10:0	31–32	3.0 × 10^−4^
Lauric acid	12:0	43–45	1.2 × 10^−5^
Myristic acid	14:0	53–58	1.0 × 10^−6^
Oleic acid	18:1; (*cis*)9	10–16	4.1 × 10^−8 c^

^a^ Values obtained from Reference [31]; ^b^ Values obtained from Reference [32]; ^c^ Value obtained from Reference [33].

**Table 3:** Summary of THCL and KTF release experiments evaluating the effect of hydrocarbon chain length of fatty acid. Data are presented as mean ± standard deviation with *n* = 3.

Contact Lens	Fatty Acid Loaded	Tetracaine Hydrochloride		Ketotifen Fumarate	
		Amount of Drug Uptake (µg/Lens)	Time for 70% Cumulative Drug Release (h)	Amount of Drug Uptake (µg/Lens)	Time for 70% Cumulative Drug Release (h)
ACUVUE TruEye	Control	120.3 ± 4.0	3.34 ± 0.21	211.1 ± 13.0	5.41 ± 1.35
	C8	46.2 ± 3.6	1.47 ± 0.14	117.9. ± 11.0	4.62 ± 0.40
	C10	140.9 ± 3.1	2.76 ± 0.79	237.7 ± 12.0	6.10 ± 0.74
	C12	356.3 ± 10.6	27.5 ± 3.56	540 ± 21.3	62.9 ± 5.66
	C14	349.2 ± 15.0	45.4 ± 3.73	516.4 ± 4.1	79.4 ± 3.55
	C18	330.6 ± 17.8	25.1 ± 1.58	502.3 ± 39.2	101.8 ± 41.4
Dailies Total	Control	99.4 ±1.7	1.86 ± 0.13	152.8 ± 7.1	2.37 ± 0.48
	C8	47.7 ± 5.0	1.73 ± 0.05	92.3. ± 13.2	2.60 ± 0.11
	C10	104.4 ± 12.3	1.79 ± 0.15	172.3 ± 3.0	1.95 ± 0.04
	C12	255.0 ± 15.3	9.90 ± 1.09	390.9 ± 26.0	24.7 ± 3.54
	C14	268.7 ± 11.1	20.0 ± 5.68	339.5 ± 15.9	22.3 ± 2.89
	C18	230.6 ± 24.5	11.1 ± 6.31	321.0 ± 23.9	21.7 ± 6.60

## Data Availability

Data is contained within the article.
